# Handbook of Diseases of the Ear, for the Use of Students and Practitioners

**Published:** 1886-12

**Authors:** 


*Handbook of Diseases of the Ear, for the use of Students and Practitioners.* By URBAN PRITCHARD, M.D. (Edin.), F.R.C.S. (Eng.) London: H. K. LEWIS.

In this excellent little work the author has "endeavoured to present a practical manual which shall help the reader to recognise the various pathological lesions of the ear, and to discriminate in the matters of treatment and prognosis;" and the result of his endeavour well fulfils the object desired. It may perhaps be doubted whether another work of this kind was required by students or practitioners; but if this be granted, then the work before us must be looked upon as eminently satisfactory. It is thoroughly practical, and is evidently the work of one who has well worked out the methods of examination described; while the treatment is simple and rational, and such as would be approved by all modern surgeons. There is an appendix of formulæ which will be very useful to the busy practitioner, and the general arrangement of the book is simple and easily understood, which cannot be said of all similar works. We can recommend the book as one of the best small works on Ear Diseases.

